# Sex steroid production associated with puberty is absent in germ cell-free salmon

**DOI:** 10.1038/s41598-017-12936-w

**Published:** 2017-10-03

**Authors:** Lene Kleppe, Eva Andersson, Kai Ove Skaftnesmo, Rolf B. Edvardsen, Per Gunnar Fjelldal, Birgitta Norberg, Jan Bogerd, Rüdiger W. Schulz, Anna Wargelius

**Affiliations:** 1 Institute of Marine Research, P.O. Box 1870, Nordnes, NO-5817 Bergen, Norway; 2 Institute of Marine Research, Matre Aquaculture Research Station, 5984 Matredal, Norway; 3 Institute of Marine Research, Austevoll Research Station, 5392 Storebø, Norway; 4 Utrecht University, Faculty of Science, Department of Biology, Padualaan 8, 3584 CH Utrecht, The Netherlands

## Abstract

In all vertebrates studied so far, germ cells are not required for pubertal maturation of the gonadal steroidogenic system, subsequent development of secondary sex characteristics and reproductive behavior. To explore if the absence of germ cells affects puberty or growth in Atlantic salmon, germ cell-free (GCF), *dnd* knockout and wild type (WT) postsmolts were stimulated to enter puberty. No GCF fish entered puberty, whereas 66.7% (males) and 30% (females) WT fish completed or entered puberty, respectively. Expression of genes related to steroidogenesis (*star*, *cyp17a1*, *cyp11β*, *cyp19a1a)*, gonadal somatic cells (*insl*3, *amh*, *igf*3), oocytes (*bmp15)*, gonadotropin receptors (*fshr*, *lhcgr)*, and pituitary gonadotropic cells (*fshb*, *lhb*, *gnrhr4*) showed an immature status and failure to up-regulate gonadal sex steroid production in male and female GCF fish was also reflected in low or undetectable plasma sex steroids (11-ketotestosterone, estradiol-17β and testosterone). A gender difference (high in females, low in males) was found in the expression of *star* and *cyp17a1* in GCF fish. No clear difference in growth was detected between GCF and immature WT fish, while growth was compromised in maturing WT males. We demonstrate for the first time in a vertebrate that germ cells are required for pubertal activation of the somatic steroidogenic cells.

## Introduction

Teleost fish share many homologies with other vertebrates, including the basic building blocks of the endocrine system regulating reproductive processes via the brain-pituitary-gonad (BPG) axis^1–3^. Considering gonadal functions at the molecular level, sex steroid hormones and growth factors regulate germ cell maturation but also exert feedback effects on the brain/pituitary level. The gonadal feedback is merged with information from other internal (e.g. nutritional or health status) and external (e.g. photoperiod, temperature, behavior of conspecifics) sources into an integrated output to control the synthesis and release of two pituitary gonadotropins, follicle-stimulating hormone (Fsh) and luteinizing hormone (Lh). However, it is uncertain how different cell types in the gonad communicate during the initiation of puberty. Germ cell-free (GCF) mice still up-regulate gonadal steroid hormone production during puberty^4^ and GCF fish display secondary sex characteristics^5–7^, suggesting that germ cells are not essential for gonadal sex steroid production and the subsequent development of secondary sex characteristics. To our knowledge, there are no studies in vertebrates showing a link between germ cells and the pubertal up-regulation of steroidogenic activity.

Gonadectomy studies in tilapia have shown that the gonads exert growth promoting effects^8^. While it is known that Sertoli cells – a somatic cell type in the testis - express growth hormone (Gh) in both tilapia and Japanese eel, studies in tilapia have shown that growth hormone receptors (*ghr*) are expressed by germ cells^8,9^, and might respond to Gh by producing Igf family members^10^. Moreover, sex steroid hormone signaling may be involved in this growth promoting effect of the gonads^8^. However, it is not clear if Gh-producing Sertoli cells or Igf-producing germ cells, or the joint activity of these cell types are responsible for the growth promoting effects of the gonads. The GCF Atlantic salmon model^11^, obtained by knocking out *dead end* (*dnd*), a well-known germ cell-specific gene in fish^11–16^, appears to be an excellently suited model to investigate the role of germ to soma communication regarding both gonadal sex steroid production and growth promoting effects.

It is yet unknown if germ cells affect both growth and maturation in salmon. To elucidate this, we induced maturation in one year old wild-type (WT) and GCF Atlantic salmon^11,17,18^, and subsequently followed growth and maturation in these fish for one full year in a common garden experiment, to study potential effects of the absence of germ cells on growth and pubertal maturation. Unexpectedly, sexual maturation was observed exclusively in WT fish, and the pubertal up-regulation sex steroid production was blocked in both female and male GCF salmon. Growth was similar in sterile mutant and immature WT fish of both sexes, but was as expected compromised in maturing WT males.

## Results and Discussion

**Figure 1:**
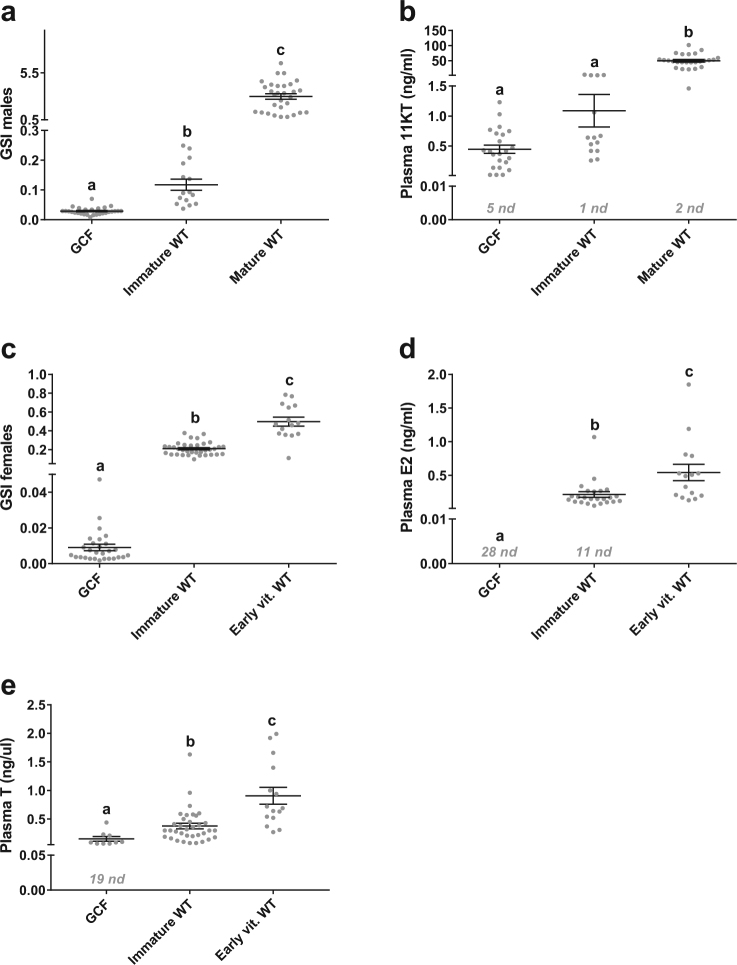
GSI and plasma sex steroid levels in GCF and WT salmon. Gonadosomatic index (GSI: (gonad weight/body weight) × 100) in Atlantic salmon males (**a**) and females (**c**), plasma 11-ketotestosterone (11-KT) in males (**b**) and plasma estradiol-17β (E_2_) (**d**) and testosterone (T) in females (**e**) at the final sampling Feb 2^nd^ 2016. Data are shown as mean with SEM, n = 28 (GCF), 35 (Immature WT females), 15 (Early vit. WT), 15 (Immature WT males) and 28–30 (Mature WT). ﻿Significant ﻿differences between groups are indicated by different letters.﻿ GCF; germ cell-free, vit; vitellogenic, WT; wild type, nd; not detected (nd = 0.01 ng/ml (11-KT and E_2_), nd = 0.05 ng/ml (T)).

Complete loss of germ cells in all vertebrates studied so far resulted in sterility, while other gonadal functions, such as sex steroid production, remain intact^4^, followed by development of secondary sex characteristics^5–7^. We have explored the phenotype of *dnd* knockout and hence GCF, sterile male salmon^11^, exposed to an established postsmolt maturation regime, usually inducing sexual maturation in 50–90% of the males in different families of Atlantic salmon^17–19^. In our study, 66.7% of the WT males (n = 45) but none of the GCF males (n = 28) were maturing in response to a maturation-inducing regime. Furthermore, we included females in the assessment of puberty. Although salmon females subjected to the postsmolt maturation regime are not recruited into the accelerated gonadal growth that is evident in males, 30% of the WT females (n = 50) responded to the maturation regime by showing a more advanced (early vitellogenic) stage of ovarian development associated with elevated GSI and plasma E_2_ levels (Fig. 1c,d). While these more advanced females may have matured earlier than females not subjected to the postsmolt maturation-inducing regime, full maturation did not occur during the experiment, in contrast to the observation made in males. Full female maturation is potentially too demanding metabolically to be triggered at this body size. In any case, none of the GCF females (n = 28) showed any sign of maturation (Fig. 1c,d).

**Figure 2:**
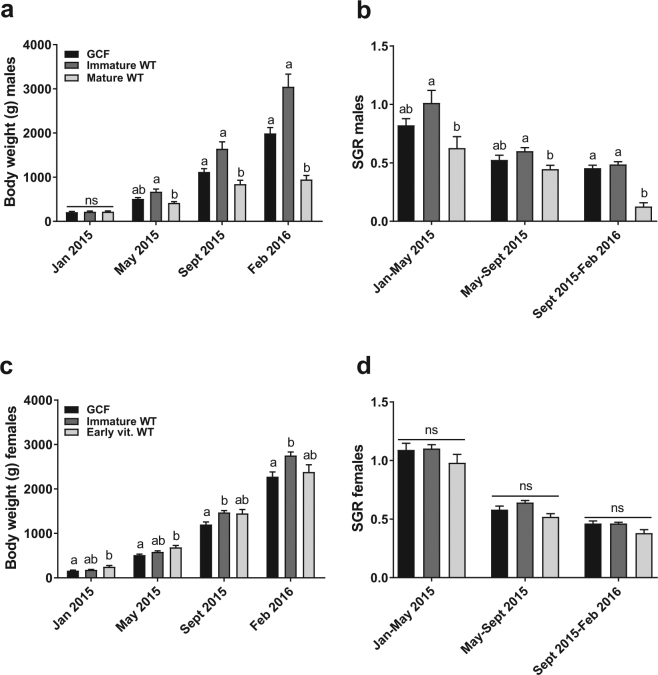
Growth of GCF and WT salmon. Growth of Atlantic salmon males (**a**,**b**) and females (**c**,**d**) shown as body weight (**a**,**c**) and specific growth rate (SGR) (**b**,**d**) throughout one year. Data are shown as mean with SEM, n = 21–28 (GCF), 34–35 (Immature WT females), 15 (Early vit. WT), 15 (Immature WT males) and 26–30 (Mature WT). Significant differences between groups are indicated by different letters. ﻿﻿ns; not significant, GCF; germ cell-free, vit; vitellogenic, WT; wild type.

In all GCF males, the plasma concentration of 11-KT was low or undetectable before the start of the maturation regime and did not increase during the experiment (Fig. 1b, Supplementary Figure S1). The low but detectable 11-KT levels in GCF males may reflect the activity of an extratesticular source, which previously has been suggested for castrated salmon^20^. While 11-KT levels did not differ between immature WT and GCF males, GSI levels were significantly higher in immature WT compared to GCF males (Fig. 1a), probably reflecting the lack of germ cells in GCF fish. It has been observed in tilapia that surgical removal of the gonads reduced somatic growth^8^. We observed a bimodal growth pattern in salmon WT males, where maturing individuals grew significantly slower than their immature counterparts (Fig. 2a,b). This finding agrees with previous observations that growth was compromised in maturing salmon^21^. GCF males grew faster than the maturing WT males and at the same rate as the immature WT group (Fig. 2a,b). The latter observations agree with findings in zebrafish, where GCF (*dnd*-knockdown) males had the same body weight as control males^22^. There was, however, a non-significant tendency of increased growth in the immature WT compared to the GCF salmon males from January to May. Investigating a larger group of fish may clarify if GCF salmon males grow slower than immature WT males.

**Figure 3:**
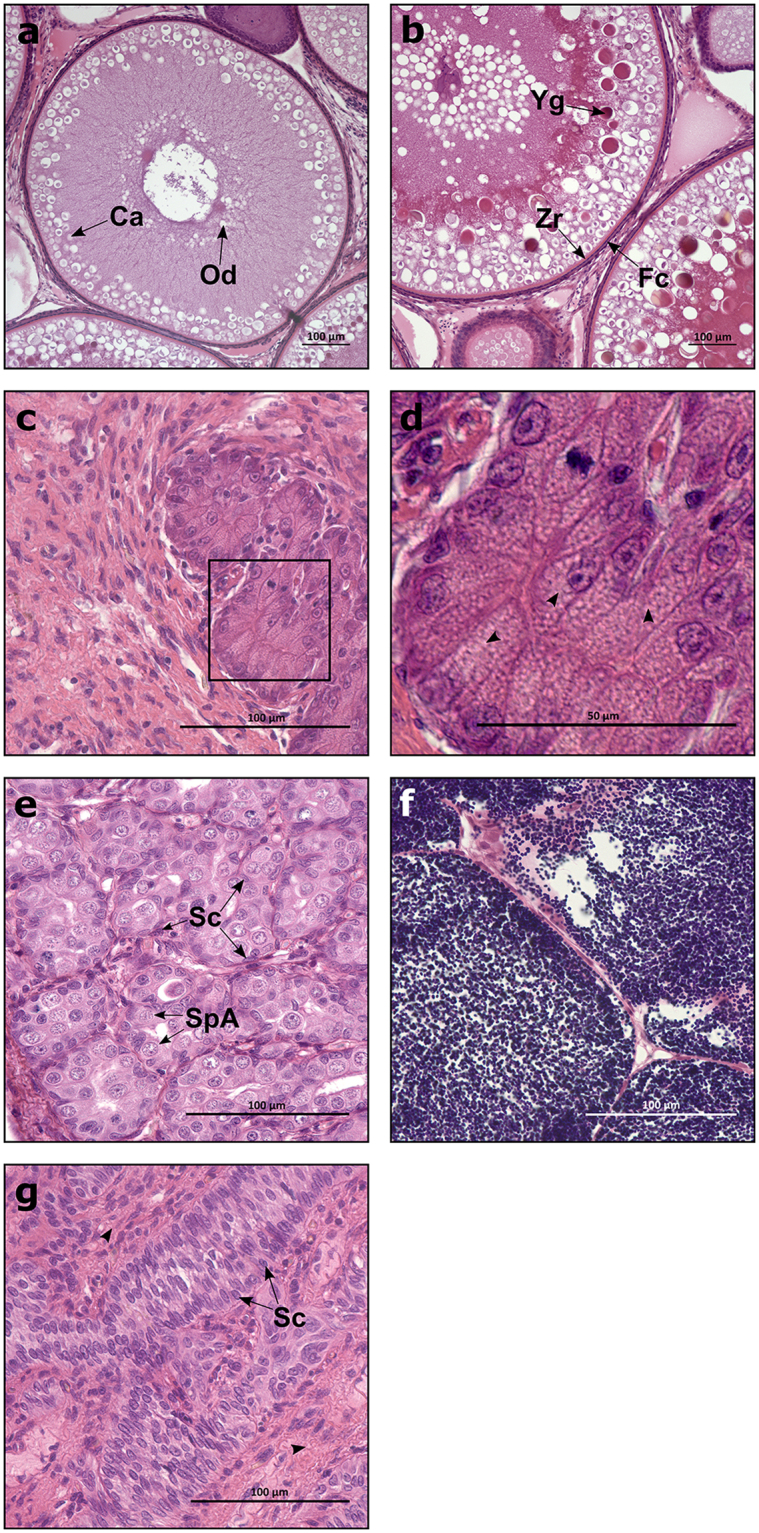
Gonadal tissue from GCF and WT salmon. Histological images of Atlantic salmon ovaries (**a**–**d**) and testis (**e**–**g**). (**a**) Immature (oildrop stage) oocytes. (**b**) Early vitellogenic oocytes. (**c**) GCF ovary. (**d**) GCF ovary with lipid vacuoles (arrowheads), magnified from the marked area in (**c**). (**e**) Immature testis. (**f**) Mature testis containing mostly spermatozoa. (**g**) GCF testis with interstitial area (arrowheads). GCF, germ cell-free; Ca, cortical alveoli; Od, oil drop; Yg, yolk granule; Zr, zona radiata; Fc, follicle cell; SpA, spermatogonia A; Sc, Sertoli cell. Scale bar = 100 µm (**a**–**c**, **e**–**g**), 50 µm (**d**).

The initiation of puberty in females is accompanied by an increase in pituitary *fshb*, plasma E_2_ and a transition from the “oildrop” immature stage to the beginning of vitellogenesis, characterized by primary yolk vesicles in the ovary^23^. Histological analysis revealed both immature (Fig. 3a; 70%) and early vitellogenic (Fig. 3b; 30%) females in the WT fish exposed to the maturation regime. In the WT group, early vitellogenic females showed higher E_2_ compared to immature animals, while both WT groups had higher E_2_ levels than the GCF females (Fig. 1d). A similar pattern was observed regarding the GSI, which was low in GCF females, higher in the immature WT group and highest in the early vitellogenic WT fish (Fig. 1c). This shows that a significant proportion of the WT females were entering puberty in response to the maturation regime, while none of the GCF females showed any signs of puberty. Histological analysis of GCF ovaries showed that the GCF stromal tissue contained cell groups with a distinct morphology (Fig. 3c,d). These cells seemed to form small glands. A single nucleolus was present in the large and round nucleus, which was oriented towards the basal compartment of the cell. Much of the cytoplasmic volume was occupied by small vacuoles (Fig. 3d). Overall, the morphology of the cells is reminiscent of hyperplastic adrenal steroidogenic cells^24^. We assume that these cells are derived from the steroidogenic theca cells in the salmon ovary.

Few studies have investigated the effects of the absence of germ cells on body growth in female fish. In Mozambique tilapia (*Oreochromis mossambicus*) females, gonadectomy retarded somatic growth^8^. Loss of germ cells due to heat treatment of Nile tilapia (*Oreochromis niloticus*) augmented growth in females, however, it was not clear whether granulosa cells had been affected or not^25^. This seems relevant because an increase in temperature induced apoptosis in granulosa cells in mammals^26^, which may result in secondary germ cell loss. In the present experiment, body weight was measured at 4 time points over a 1.5-year period, with fish size increasing from around 50 to 2500 g. The early vitellogenic WT were heavier than the GCF females at the start of the experiment (Fig. 2c). Over time, this weight difference disappeared, although the immature WT group became heavier than GCF females (Fig. 2c). Nevertheless, no significant differences were detected in SGR between any female groups at any time point in this study (Fig. 2d), suggesting that there are no negative effects on growth in GCF salmon females. Since we could not follow the female fish into full maturity in the present study, we cannot exclude a growth effect at later stages. However, in commercial production, females are harvested before rapid ovarian growth starts. Therefore, we do not expect a negative effect on growth due to loss of germ cells.

**Figure 4:**
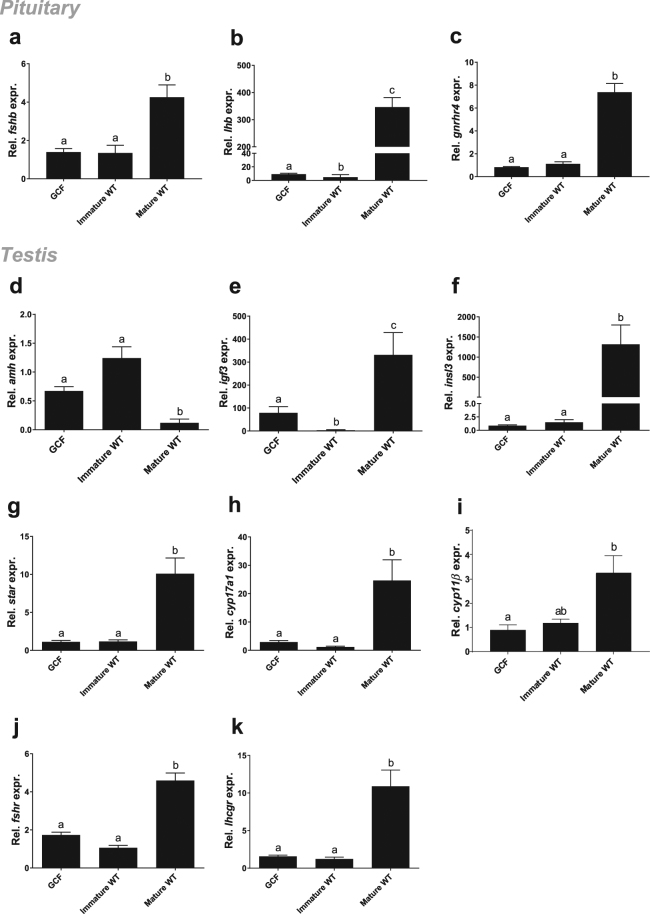
Gene expression in pituitary and testis from GCF and WT males. Expression of *fshb* (**a**), *lhb* (**b**), *gnrhr4* (**c**), *amh* (**d**), *igf3* (**e**), *insl3* (**f**), *star* (**g**), *cyp17a1* (**h**), *cyp11β* (**i**), *fshr* (**j**), and *lhcgr* (**k**) relative to *ef1a*, in Atlantic salmon male pituitaries (**a**–**c**) and testes (**d**–**k**), measured by qPCR. Data are shown as mean with SEM, n = 21–22 (GCF), 8–10 (Immature WT) and 8–9 (Mature WT). Significant differences between groups﻿ are indicated by different letters. GCF; germ cell-free, WT; wild type.

What molecular signature accompanies the inability of gonadal tissue in GCF fish to activate sex steroid production? To pursue this question, we analysed the expression of selected genes in both gonad and pituitary samples from GCF and WT fish. In testis tissue, we measured transcript levels of *anti-müllerian hormone* (*amh*, Fig. 4d) and *insulin-like growth factor 3* (*igf3*, Fig. 4e), as these are Sertoli cell factors and markers of immature and maturing testis, respectively^27^. In GCF testis, *amh* was expressed at the same level as in immature WT males, indicating an immature status of the Sertoli cells in the GCF testis (Fig. 4d). The *igf3* transcript levels were significantly higher in GCF testis compared to immature WT, but lower than in mature WT (Fig. 4e). This may indicate that Sertoli cells in GCF males show some degree of maturation. Sertoli cells were rather abundant in GCF testis and Sertoli cell number in the GCF tubuli seemed to increase with time, comparing the morphology of the tubuli between 1 and 2.5-year-old GCF males (Fig. 3g ^11^). Since Igf3 also stimulated Sertoli cell proliferation in zebrafish^28^, elevated Igf3 signalling might contribute to an autocrine loop stimulating Sertoli cell proliferation, leading to Sertoli cell-rich but germ cell-free tubuli (Fig. 3g). Up-regulation of *igf3* in maturing compared to immature WT males seems to be in line with reports in zebrafish that Fsh-stimulated Igf3 release by Sertoli cells promotes spermatogenesis^29^. Further, we measured the expression of *insulin-like peptide 3* (*insl3*), which is a Leydig cell-derived stimulator of spermatogonial differentiation in zebrafish^30^. Moreover, *insl3* transcript levels are exquisitely sensitive to Fsh in zebrafish^31^. The transcript level of *insl3* in GCF testes was low and not different from immature WT, but was up-regulated in mature WT testes. This suggests that both immature and GCF males experience low Fsh stimulation, in strong contrast to mature males (Fig. 4f).

To investigate if changes in the expression of steroidogenesis-related genes could explain the low 11-KT plasma levels in GCF males, we measured *steroidogenic acute regulatory protein* (*star*), *cytochrome p450 17a1* (*cyp17a1*) and *11-beta-hydroxylase* (*cyp11β*) transcript levels in testis tissue. StAR facilitates the rapid, gonadotropin-stimulated translocation of cholesterol to the inner mitochondrial membrane in steroidogenic cells, where the rate-limiting step of steroid production takes place, cholesterol side-chain cleavage^32,33^. Cyp17a is involved in converting i) pregnenolone to 17-hydroxypregnenolone, ii) progesterone to 17α-hydroxyprogesterone and iii) 17α-hydroxyprogesterone to androstenedione^34^, an essential step towards androgen production. Cyp11β is essential for the conversion of testosterone but predominantly androstenedione^35^ to 11beta-hydroxylated androgens^36^. The transcript level of *star* was similarly low in GCF and immature males but clearly up-regulated in mature WT testis tissue (Fig. 4g). The changes in *cyp17a1* and *cyp11β* transcript levels showed patterns similar to that of *star* (Fig. 4h,i). The lower levels of *star*, *cyp17a1* and *cyp11β* suggest that expression of two key genes in androgen biosynthesis, and the gene required for 11beta-hydroxylation of androgens, failed to be up-regulated in GCF testis. This is in line with low 11-KT plasma levels found in GCF males. Moreover, analysis of pituitary transcripts *fshb*, *lhb* and *gnrhr4* revealed low levels in GCF and immature males, which became significantly up-regulated in mature WT males (Fig. 4a–c). The same pattern emerged from quantifying *fshr* and *lhcgr* transcripts in testis tissue (Fig. 4j,k). At present, we have no explanation for the significantly lower *lhb* expression in the pituitary of immature WT compared to GCF fish (Fig. 4b). Taken together, this data set suggests that GCF testes fail to produce a signal that seems required to enable the brain-pituitary system to respond to the stimulatory photoperiod/temperature cues. Consequently, pituitary sensitivity to Gnrh and testicular sensitivity to gonadotropins remain in a prepubertal status. In the absence of data on circulating gonadotropins, we can infer from our data on testicular transcript levels that a gonadotropic stimulation did not occur in GCF males. In this regard, it will be interesting to study in future experiments for example, if the GCF testis can respond to exogenous gonadotropin.

**Figure 5:**
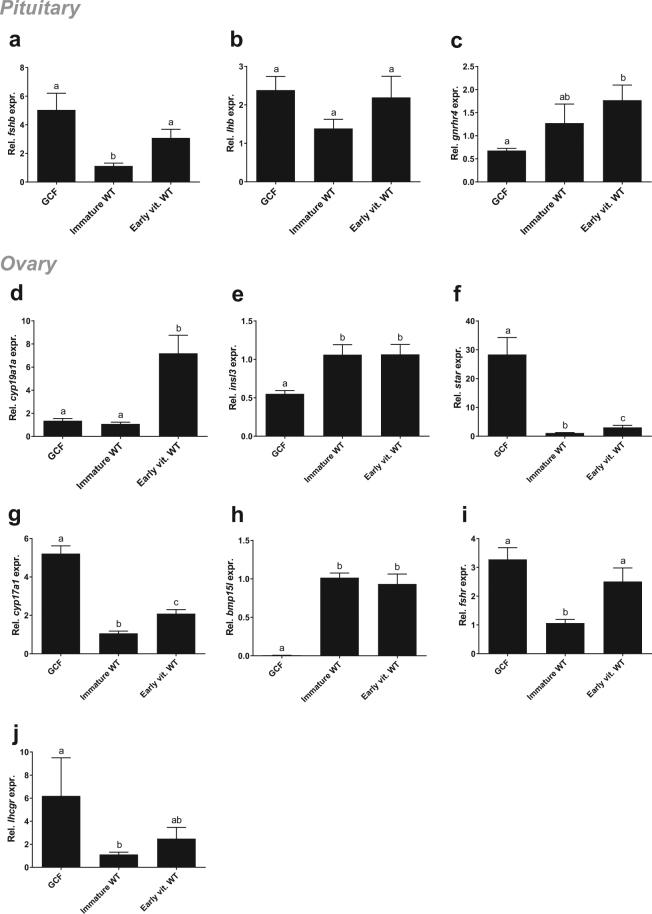
Gene expression in pituitary and ovary from GCF and WT females. Expression of *fshb* (**a**), *lhb* (**b**), *gnrhr4* (**c**), *cyp19a1* (**d**), *insl3* (**e**), *star* (**f**), *cyp17a1* (**g**), *bmp15l* (**h**), *fshr* (**i**) and *lhcgr* (**j**) relative to *ef1a*, in Atlantic salmon female pituitaries (**a**–**c**) and ovaries (**d**–**j**), measured by qPCR. Data are shown as mean with SEM, n = 21–24 (GCF), 9–10 (Immature WT) and 8–10 (Vitellogenic WT). Significant differences between groups are indicated by different letters. GCF; germ cell-free, vit; vitellogenic, WT; wild type.

In ovarian tissue, we measured the expression of selected genes expressed by somatic cells, including granulosa cells, *aromatase* (*cyp19a1a*), required for estrogen production in the ovary^37^. As expected (Fig. 1d), *cyp19a1a* was significantly higher expressed in early vitellogenic WT ovaries compared to both GCF and immature WT ovaries (Fig. 5d), indicating an immature status of the GCF ovary. Furthermore, we measured the ovarian mRNA level of *insl3*, which was significantly lower in GCF ovaries compared to both immature and early vitellogenic WT ovaries (Fig. 5e). Limited information exists on Insl3 function in fish ovaries, however, in mammals INSL3 may have a regulatory role in maintaining thecal androgen production^38^. In contrast to the GCF testis, the GCF ovary displayed clearly elevated transcript levels of *star* and *cyp17a1*, suggesting that substrate availability for the cholesterol side-chain cleavage and the production of androstenedione were not limiting factors (Fig. 5f,g), implying that an initial and an intermediate step of the steroidogenic pathway towards estrogen production were not inhibited in GCF females. We were not able to measure androstenedione, which is a precursor of both testosterone and estrogen, however, we were able to measure testosterone that, similar to E_2_ (Fig. 1d), was found to be low or undetected in GCF females (Fig. 1e). Elevated transcript levels of *fshb* (Fig. 5a) and its receptor *fshr* (Fig. 5i), in conjunction with elevated levels of steroidogenesis-related genes *star* and *cyp17a1* (Fig. 5f,g) would all be in line with a high level of gonadotropic stimulation of the GCF ovaries. Female mice devoid of germ cells due to knockout of *dazl*, showed increased levels of plasma Fsh^4^, pointing at a conserved trait between salmon and mouse. Moreover, elevated circulating gonadotropin levels are characteristic of postmenopausal primates, where the germ cell-depleted ovary is unable to sustain estrogen production^39^. In coho salmon, Fsh stimulated the expression of *star* and *cyp17a1*, while *cyp19a1a* remained unaffected^40^. Likewise, we observed no upregulation of *cyp19a1a* in our mutants compared to immature WT fish. Although Fsh up-regulated E_2_ release via a cAMP-dependent mechanism in brook trout^41^, this does not necessarily involve an increase in *cyp19a1a* transcript levels. In our study, the very low E_2_ plasma levels in GCF salmon females suggest that the potentially elevated circulating Fsh levels could neither induce a noticeable E_2_ production, probably due to the very low aromatase activity, nor that an up-regulation of *cyp19a1a* took place. In the GCF ovary, glandular structures were observed that were composed of cells showing small lipid vacuoles in their cytoplasm (Fig. 3c,d), a feature typical of steroidogenic cells. However, the high number of these vacuoles is unusual and may reflect a strong gonadotropic stimulation. An accumulation of these lipid droplets has also been observed in steroidogenic cells of the adrenal cortex in genetically modified mice showing a high level of ACTH stimulation^42^. However, in this model the *star* gene was missing while GCF ovaries show elevated *star* transcript levels that despite the also elevated *cyp17a1* transcript levels, still did not result in elevated androgen production. Hence, unravelling the exact mechanism responsible for the steroidogenic failure in the GCF ovary requires further research.

The data presented so far suggest that in GCF ovaries, part of the steroidogenic system operates at an elevated level of activity, contrasted by low ovarian aromatase expression and very low E_2_ plasma levels. Considering germ to somatic cell signaling, however, a possible explanation for low *cyp19a1a* transcript levels could be the lack of communication, for example *via* the oocyte-derived Tgf-β family member Bone morphogenetic protein 15 (Bmp15) to surrounding somatic cells. In zebrafish ovaries, loss of Bmp15 resulted in a failure to up-regulate *cyp19a1a* transcript levels in granulosa cells^43^. In GCF salmon ovaries, the transcript level of *bmp15l* was drastically reduced compared to both immature and early vitellogenic WT ovaries (Fig. 5h), suggesting that the germ cells are the main source of this growth factor in salmon ovaries. Thus, the strong reduction in Bmp15 signalling in GCF ovaries may be responsible for the failure to up-regulate *cyp19a1a* expression, as shown in zebrafish^43^. These authors moreover suggested that the remaining *cyp19a1a* expression in *bmp15* ^−/−^ zebrafish mutants is located in thecal cells, which may have aggregated in the gland-like structures found in the GCF ovaries. Expression of *lhb* in female pituitaries did not differ between any groups (Fig. 5b), which suggests that there is no feedback mechanism from the GCF ovary. The *lhcgr* expression in ovaries showed a similar profile, although the GCF group had significantly higher expression than the immature WT group (Fig. 5j). Nevertheless, the GCF group showed a high variation, which calls for a higher n to conclude. Pituitary transcript level of *gnrhr4* in females was lowest in the GCF group, and significantly higher in the early vitellogenic group (Fig. 5c). Since the presence of sex steroids are involved in the regulation of *gnrhr4* ^18,44^, it is expected that the GCF group, which had low or undetectable levels of E_2_ (Fig. 1d) and T (Fig. 1e), would have the lowest level of *gnrhr4*. Taken together, the levels of molecular markers measured in GCF females in this study support that these animals are immature, and that the steroidogenic pathway is inhibited.

There is a clear gender difference in the size and content of gonadal somatic cells, in addition to the presence (males) or absence (females) of the *sdY* gene^45,46^, in GCF salmon. The GCF testis showed a high number of Sertoli cells, while the GCF ovary has almost no distinguishable follicular cells in 2.5 years old females, although granulosa-like cells were observed in 1 year old GCF females^11^. This finding implies a sex-dependent difference in the capacity of the GCF gonad to adhere to its morphogenetic program: the GCF testis still shows spermatogenic tubuli though they are devoid of germ cells and instead filled by Sertoli cells; also, the interstitial area shows no obvious deviations from wild-type immature testicular interstitium. In the GCF ovary, on the other hand, somatic structures like the thecal and granulosa layers evidently cannot form and instead glandular structures appear that are not known from wild type ovarian tissue. The latter, however, may represent an agglomeration of the special theca cells. Hence, in both sexes, the principle steroidogenic cells, special theca and Leydig cells, seem in their cellular identity to be independent of the presence of germ cells. Despite the survival of steroidogenic cells in both sexes, the up-regulation of sex steroid production usually observed during puberty fails to occur in males and is also missing with respect to E_2_ production in females. However, there are also sex-dependent differences in the response of the steroidogenic system to the GCF status. Finally, we observed no clear effect on body growth in GCF males or females, compared to their immature WT counterparts, while both showed a clear growth advantage compared to maturing wild type males.

Our study shows for the first time in a vertebrate, the Atlantic salmon, that the pubertal activation of gonadal androgen and estrogen production in testis and ovary requires the presence of germ cells. Furthermore, we demonstrate that in the absence of germ cells, there is a gender difference in the expression of some of the key genes (*star* and *cyp17a1*) involved in steroidogenesis. Future studies should investigate the molecular mechanisms that are causing the inhibition of steroidogenesis. Moreover, this study confirms our previous finding that the sex differentiation process is comparatively stable in salmon, compared to other fishes, since neither loss of germ cells nor the clear reduction in sex steroid release and the loss of recognizable somatic structures in the GCF ovary lead to a female to male sex change. Finally, body growth is not impaired by the loss of germ cells in salmon.

## Materials and Methods

### Experimental Design

The fish used in this study were reared and sampled at Matre Aquaculture Research Station, Matredal, Norway. GCF (*dnd* knockout) and WT fish were produced as described in Wargelius *et al*.^11^. The eggs used in the experiment were fertilized in Nov 2014, and juveniles were fed from April 2014 and reared under standard conditions until Oct 2014. From Oct 2^nd^ 2014 fish were exposed to a 12 hours dark and 12 hours light regime for a period of 7 weeks, to ensure smoltification. Oct 23^rd^ all fish were pit tagged and placed in common garden tanks with equal amounts of GCF and WT in each tank (11 mm Trovan ID 101 tags, BTS Scandinavia AB, Sweden). Nov 18^th^ the fish were exposed to a continuous light regime and 16 °C to induce postsmolt maturation for a period of 4 months^17^. Subsequently, fish were transferred to sea water and kept at ambient temperature (~9 °C) and natural photoperiod until termination of the experiment (Feb 2^nd^ 2016). Feeding was done with standard commercial diets. Prior to sampling for length, weight and plasma (Jan 20^th^ (no plasma sampling), May 5^th^ and Sept 25^th^ 2015), the fish were anesthetized with 1 ml/L finquel vet. At the final sampling (Feb 2^nd^ 2016), all fish were anesthetized with 2 ml/L finquel vet and sacrificed by cutting into the *medulla oblongata* (after sampling of blood). From the sampled fish, one complete gonad and the pituitary were immediately frozen in liquid nitrogen, and stored at −80 °C until further use for RNA isolation and subsequent qPCR analysis. The other gonad was fixed in Bouin’s fixative for 6–24 hours, then transferred into 70% ethanol, and further dehydrated and embedded in paraffin using a Histokinette (Leica TP1020).

### Real-time, Quantitative PCR Assays

RNA was extracted from gonad and pituitary tissue using the iPrep™ Trizol® Plus RNA Kit (Thermo Fischer Scientific), according to the manufacturer’s instructions. Purified RNA was then treated with the turbo DNA-free kit (Ambion) to remove trace amounts of contaminating DNA. 500 ng of treated RNA served as input for reverse transcription reaction using the VILO cDNA synthesis kit (Invitrogen) for each sample. Previously published primers and hydrolysis probes specific for Atlantic salmon *fshb*, *lhb* ^23^, *gnrhr4* ^18^, *fshr*, *lhcgr* ^47^, *igf3*, *amh* ^27^, *cyp19a1a* ^11^ and *bmp15l* ^48^ were used in this study. Furthermore, primers and probe sequences for *cyp17a1*, *star* and *cyp11β* were designed online (http://probes.pw.usda.gov/batchprimer3/) and are listed in Supplementary Table S1. The primer sequences for *insl3* (Supplementary Table S1) were designed using Primer Express 3.0 (Applied Biosystems); cloning and sequencing of salmon *insl3* is described in Supplementary Note S1.

All qPCR assays were performed in duplicates, using 384-well optical plates on an ABI Prism 7700 Sequence Detection System (Applied Biosystems) (male pituitaries) or QuantStudio 5 Real-Time PCR System (ThermoFisher Scientific) (testis, ovaries and female pituitaries) using default settings. One µl of a 1/20 (gonads) or 1/40 dilution (pituitaries) of cDNA was used in a 10 µl Fast Taqman qPCR reaction (ThermoFisher Scientific). In the case of *star*, *cyp17a1*, *cyp11β* and *insl3*, 1 µl of a 1/160 dilution of cDNA was used in a 10 µl Fast SYBR green qPCR reaction (ThermoFisher Scientific). For each qPCR assay, melting-curve analysis showed that only one product was generated. For each PCR plate, no-template controls were run for each gene. The relative gene expression levels for all genes were calculated using the comparative Ct (or 2^−ΔΔCt^) method. For each gene, all values were normalized to *ef1a* and calibrated to the average ΔCt of the immature WT group.

### Gonadosomatic Index and Specific Growth Rate

Gonadosomatic index (GSI) was determined as: GSI (%) = gonad weight (g) * 100/total body weight (g). Specific growth rate (SGR) was calculated as: SGR (%) = 100 * ((ln body weight_start_ − ln body weight_end_)/number of days.

### Steroid Hormone Quantification

Sex steroids (11-ketotestosterone: 11-KT, testosterone: T, and estradiol-17β: E_2_) were analyzed with ELISA^49^ and validated for Atlantic salmon, on extracted plasma samples as detailed previously^23^.

### Determination of Sex and Scoring of Sexual Maturity

To determine the sex of each GCF fish, the presence (males) or absence (females) of the sex determining gene *sdY* ^45,46^ was measured by PCR^11^. For the scoring of sexual maturity, all gonads were embedded in paraffin, sectioned and stained with hematoxylin, eosine and saffron (HES). The females were scored as immature (oildrop stage) or early vitellogenic based on histology (Fig. 3a,b), GSI and level of estradiol-17β (E_2_) (Supplementary Table S2). The males were scored as immature (Fig. 3e) or mature (Fig. 3f) based on GSI and level of 11-ketotestosterone (11-KT) (Supplementary Table S2).

### Statistics

Statistical tests were performed using GraphPad Prism 7.02 (GraphPad Software Inc.). All datasets were tested for normal distribution using a D’Agostino & Pearson omnibus normality test. A difference between groups was considered significant when P < 0.05. A nonparametric Kruskal-Wallis with Dunn’s multiple comparisons test was applied to test for significant differences in the following datasets: GSI, plasma steroids, body weight and SGR. For the qPCR datasets, all values were log-transformed to obtain a more normal distribution. In cases where all groups to be compared passed the normality test, a one-way ANOVA with Tukey’s multiple comparisons test was applied on log-values to identify significant differences. In cases where one or more of the groups to be compared did not pass the normality test, we applied Kruskal-Wallis with Dunn’s multiple comparisons test on gene expression values.

### Use of Experimental Animals

This experiment was approved by the Norwegian Animal Research Authority (NARA, permit number 5741) and the use of these experimental animals was in accordance with the Norwegian Animal Welfare Act.

### Data Availability

The datasets generated and analysed during the current study are included in this published article (and its Supplementary Information Files).

## Electronic supplementary material

Supplementary Information
