# Supplementary material for: Sex steroid production associated with puberty is absent in germ cell-free salmon
Source: Sci Rep. 2017 Oct 3;7:12584. doi: 10.1038/s41598-017-12936-w (PMC5626747; doi:10.1038/s41598-017-12936-w)
Supplement: Supplementary file 1 — Supplementary Information [file 41598_2017_12936_MOESM1_ESM.pdf]

# Supplementary Information for the article;

# *Sex steroid production associated with puberty is absent in germ cell-free salmon*

Lene Kleppe[1*], Eva Andersson[1], Kai Ove Skaftnesmo[1], Rolf Brudvik Edvardsen[1], Per Gunnar Fjelldal[2], Birgitta Norberg[3], Jan Bogerd[4], Rüdiger W. Schulz[1,4], Anna Wargelius[1]

[1]Institute of Marine Research, P.O. Box 1870, Nordnes, NO-5817 Bergen, Norway
[2]Institute of Marine Research, Matre Aquaculture Research Station, 5984 Matredal, Norway
[3]Institute of Marine Research, Austevoll Research Station, 5392 Storebø, Norway
[4]Utrecht University, Faculty of Science, Department of Biology, Padualaan 8, 3584 CH Utrecht, The Netherlands

Contact:
*Corresponding author, lene.kleppe@imr.no

## Supplementary Figure S1.

Plasma sex steroid levels in males (11-KT) and females ($E_2$) at 3 samplings from May 2015 to February 2016. Data are shown as mean with SEM. N = 12-28 (males) and (14-35) females. A Kruskal-Wallis test with Dunn's post-test was applied to reveal significant differences between time points for each group of fish. Different letters within a group of fish represent significant (p < 0,05) differences. GCF; germ cell-free, vit; vitellogenic, WT; wild type, ns; not significant.

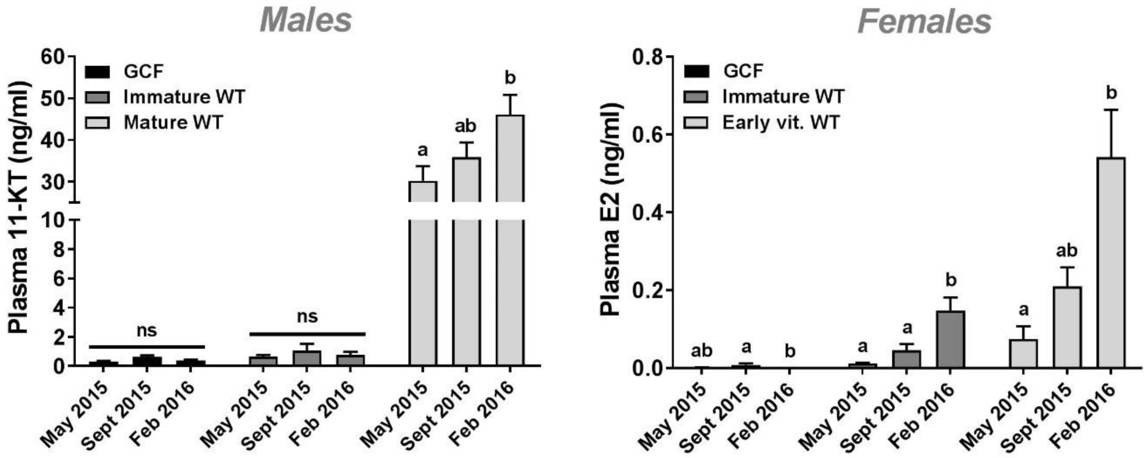

# Supplementary Note S1.

## Cloning of Atlantic salmon *insl3* cDNA sequence

In the official Atlantic salmon genome annotation (NCBI *Salmo salar* Annotation Release 100), the *insl3* gene is not annotated. Therefore, the *Danio rerio insl3* cDNA sequence (GenBank acc. no. EF685704) was blasted against the salmon genome to identify the potential coding sequence of salmon *insl3*. A putative *insl3* sequence was found in chromosome ssa14: 12666199-12663739. Primers 3534-3538 (**Table 1**) were designed in order to specifically amplify the predicted coding region of the *Salmo salar insl3* cDNA. PCR products were obtained between primers 3534 and 3537, primers 3535 and 3537, and primers 3536 and 3538, gel purified, cloned and sequenced. Based on these sequences, new primers were designed and used to obtain 3'-RACE (primer 3591 in combination with the universal primer mix [UPM] and primer 3602 in combination with the nested universal primer [NUP]) and 5'-RACE (primer 3558 in combination with UPM and primer 3559 in combination with NUP) products with the SMARTER RACE cDNA amplification kit (Clontech, Mountain View, CA, USA), according to the manufacturer's recommendations. RACE products were gel purified, cloned and sequenced. The sequences of the 5'-RACE and 3'-RACE products, and the initial RT-PCR product, were combined to a single consensus Atlantic salmon *insl3* cDNA sequence of 857 nucleotides, which was deposited to GenBank (acc. no. MF062497), and codes for a precursor protein of 159 amino acids (**Fig. 1**).

**Table 1.** Primers used in this study.

| Primer name | Primer sequence (5' → 3') |
|---|---|
| 3534 | CGCTGCATCGATTTGCTGTTTGTGTG |
| 3535 | GGGCGCAAACACCCTCATCCAACATC |
| 3536 | ATGGATGCTAAGTGTCTTGTGTCTCTTGTGGTAGTTCTC |
| 3537 | CCAACAATTCCTTGTGAAATGTTACTCTTGCCTAG |
| 3538 | GCTTAGCAATACTGGACCAGTTCGCTCATGG |
| 3558 | AATCCATCCTTGCAATGGCTGACCAACAATTC |
| 3559 | GTGCAGCCGGATCGGCAGCATACC |
| 3591 | CCCATCCAGGGTTTCCCAGCACA |
| 3602 | CCAAGTGGACATGGAGCCTTCAACCGTCC |

```
ACACATCACATCCCTCCTCACCTCATCGTACCATAC          36
TCGCTGCATCGATTTGCTGTTTGTGTGATTTACTTGGGCGCAGACACCCTCATCAACATC      96

 M  D  A  K  C  L  V  S  L  V  V  V  L  M  V  G  V  Y  G  T
ATGGATGCTAAGTGTCTTGTGTCTCTTGTGGTAGTTCTCATGGTTGGGGTGTATGGGACC     156

 H  G ▼Q  D  A  R  V  K  L  C  G  R  E  F  I  R  M  V  V  T
CACGGGCAGGATGCCAGGGTGAAGCTGTGTGGGAGAGAGTTTATCCGTATGGTAGTCACA     216

 S  C  G  S  S  R  L  K  R  S  T  P  E  L  G  Q  H  P  V  N
TCGTGTGGGAGCTCCAGACTGAAGCGCTCCACTCCGGAGCTTGGACAACACCCCGTCAAC     276

 H  H  R  V  I  L  D  W  L  N  S  N  Q  F  A  N  L  R  T  S
CACCACAGGGTAATTCTGGATTGGCTGAACAGCAATCAATTTGCCAACCTGAGGACTTCA     336

 E  T  G  E  D  V  V  T  Q  Q  D  D  Q  D  S  T  E  S  M  T
GAGACTGGTGAAGATGTGGTGACACAGCAAGATGACCAGGACTCCACAGAGAGCATGACA     396
```

```
 E  D  Q  Q  T  H  P  G  F  P  S  T  P  P  Q  V  Q  V  D  M
GAAGACCAGCAGACCCATCCAGGGTTTCCCAGCACACCTCCACAGGTCCAAGTGGACATG     456

 E  P  S  T  V  P  L  G  Y  T  V  S  S  R  T  R  R  D  V  G
GAGCCTTCAACCGTCCCTCTGGGATACACCGTGTCCTCCCGTACCCGCCGAGACGTGGGG     516

 P  A  G  V  C  C  R  S  G  C  T  M  S  E  L  V  Q  Y  C  *
CCTGCTGGGGTATGCTGCCGATCCGGCTGCACCATGAGCGAACTGGTCCAGTATTGCTAA     576
GCTATAGCTAATGCTAGGCAAGAGTAACATTTCACAAGGAATTGTTGGTCAGCCATTGCA     636
AGGATGGATTAGGTTAAAATCAGGCTTATTTACATTTGTTGGGATGTTTGAAGGGCCCCA     696
TTTTGCAGTTGCCAAACCTTTGCATTAGAGCCAATGCAGTTAGTTCTACTGAAATGTTTT     756
ACTAAAATGTTCATGTTGTTTTCTTGATATCATGAATTGTGTGTTAATCTAATCGTTAGA     816
ATATTGTTGCGGAATGACTTGTGTAATGACAAAATCAAGAC-poly A                 857
```

**Figure 1. Nucleotide sequence of the Atlantic salmon *insl3* cDNA as well as the deduced amino acid sequence of the Atlantic salmon Insl3 precursor.** Nucleotides are numbered 5'- to 3'. The first residue of the initiation codon of the coding region is nucleotide 97, while the stop codon (indicated with the asterisk) consists of nucleotides 574-576. The potential cleavage site between the signal sequence and the Insl3 prohormone is indicated by the downward arrowhead. The Insl3 precursor protein consists of the B chain (indicated in with a blue background), followed by a proteolytic cleavage site (black background), the C peptide (yellow background), a second proteolytic cleavage site (black background) and the A chain (green background). Cysteine residues (indicated with a red background) in the B- and A-chains form two interchain disulfide bridges as well as one intrachain disulfide bridge.

## Supplementary Table S1.

Primer sequences for Atlantic salmon *steroid 17-alpha-hydroxylase/17,20 lyase* (*cyp17a1*), *steroidogenic acute regulatory protein* (*star*), *11-beta-hydroxylase* (*cyp11β*) and *insulin-like peptide 3* (*insl3*).

| **Gene** | **Forward primer (5'-3')** | **Reverse primer (5'-3')** |
|---|---|---|
| *star* (GenBank acc. no: 100136492) | ATGACCCCAACAAGACCAAG | GGGATCCAGCCCTTTAAATC |
| *cyp17a1* (GenBank acc. no: 106576693) | TCCCATGGCTACAGGTCTTC | CTGCTTTAGGAGACGCAGGT |
| *cyp11β* (GenBank acc. no: 100136945) | CGAAATGCAGCTGCTACTGA | AGGCTGGAGGATTAGGGTGT |
| *insl3* (GenBank acc. no: MF062497) | CTCCGGAGCTTGGACAACAC | AGTCCTCAGGTTGGCAAATTGAT |

## Supplementary Table S2.

Overview of all the fish studied at end sampling 02.02.2016. For each fish the sample number, sex, maturation stage, gonadosomatic index (GSI) and level of plasma sex steroid ($E_2$, T or 11-KT) are shown. vit; vitellogenic, n.d; not detected (n.d. = 0.01 ng/ml (11-KT and $E_2$), n.d. = 0.05 ng/ml (T)).

| Sample | Sex | Maturation stage | GSI | 11-KT (ng/ml) | $E_2$ (ng/ml) | T (ng/ml) |
|---|---|---|---|---|---|---|
| 12 | Female | GCF | 0,0062 | | n.d. | n.d. |
| 13 | | | 0,0076 | | n.d. | n.d. |
| 24 | | | 0,0137 | | n.d. | n.d. |
| 28 | | | 0,0027 | | n.d. | n.d. |
| 93 | | | 0,0036 | | n.d. | n.d. |
| 118 | | | 0,0028 | | n.d. | n.d. |
| 119 | | | 0,0033 | | n.d. | 0,21 |
| 163 | | | 0,0027 | | n.d. | n.d. |
| 166 | | | 0,0037 | | n.d. | n.d. |
| 167 | | | 0,0255 | | n.d. | 0,1 |
| 171 | | | 0,0027 | | n.d. | n.d. |
| 186 | | | 0,0197 | | n.d. | n.d. |
| 44 | | | 0,0091 | | n.d. | n.d. |
| 48 | | | 0,0071 | | n.d. | n.d. |
| 80 | | | 0,0094 | | n.d. | n.d. |
| 164 | | | 0,0036 | | n.d. | n.d. |
| 174 | | | 0,0028 | | n.d. | n.d. |
| 176 | | | 0,0156 | | n.d. | 0,09 |
| 43 | | | 0,0057 | | n.d. | n.d. |
| 45 | | | 0,0078 | | n.d. | 0,07 |
| 46 | | | 0,0111 | | n.d. | 0,09 |
| 47 | | | 0,0114 | | n.d. | n.d. |
| 84 | | | 0,0472 | | n.d. | n.d. |
| 168 | | | 0,0048 | | n.d. | 0,07 |
| 182 | | | 0,0141 | | n.d. | 0,44 |
| 151 | | | 0,0037 | | n.d. | 0,23 |
| 26 | | | 0,0047 | | n.d. | 0,07 |
| 87 | | | 0,0018 | | n.d. | n.d. |
| 30 | Female | Immature WT | 0,1749 | | 0,27 | 0,41 |
| 31 | | | 0,2004 | | 0,1 | 0,3 |
| 32 | | | 0,3222 | | 0,45 | 0,96 |
| 35 | | | 0,2083 | | 1,07 | 1,63 |
| 39 | | | 0,2088 | | 0,15 | 0,56 |
| 40 | | | 0,2331 | | 0,11 | 0,25 |
| 41 | | | 0,1403 | | n.d. | 0,16 |
| 42 | | | 0,1472 | | n.d. | 0,12 |
| 53 | | | 0,1533 | | n.d. | 0,22 |
| 133 | | | 0,3288 | | 0,11 | 0,57 |
| 134 | | | 0,1643 | | 0,08 | 0,21 |
| 137 | | | 0,1729 | | 0,15 | 0,32 |
| 145 | | | 0,1410 | | n.d. | 0,38 |
| 147 | | | 0,1952 | | 0,15 | 0,73 |
| 154 | | | 0,1396 | | n.d. | 0,14 |
| 2 | | | 0,2269 | | n.d. | 0,3 |

| | | | | | | |
|---|---|---|---|---|---|---|
| 5 | | | 0,3661 | | 0,1 | 0,3 |
| 17 | | | 0,1473 | | 0,14 | 0,3 |
| 27 | | | 0,1363 | | n.d. | 0,08 |
| 63 | | | 0,0976 | | n.d. | 0,08 |
| 95 | | | 0,1914 | | 0,08 | 0,19 |
| 108 | | | 0,2663 | | 0,19 | 0,36 |
| 112 | | | 0,1681 | | 0,18 | 0,5 |
| 115 | | | 0,2464 | | 0,29 | 0,58 |
| 123 | | | 0,2296 | | 0,19 | 0,6 |
| 161 | | | 0,2501 | | 0,27 | 0,28 |
| 165 | | | 0,1827 | | n.d. | 0,18 |
| 175 | | | 0,2241 | | 0,17 | 0,25 |
| 177 | | | 0,2423 | | n.d. | 0,2 |
| 180 | | | 0,1455 | | n.d. | 0,1 |
| 187 | | | 0,1477 | | n.d. | 0,1 |
| 188 | | | 0,3758 | | 0,26 | 0,59 |
| 72 | | | 0,2259 | | 0,17 | 0,43 |
| 73 | | | 0,2624 | | 0,34 | 0,45 |
| 83 | | | 0,2774 | | 0,12 | 0,39 |
| 52 | | | 0,3672 | | 0,79 | 1,4 |
| 129 | | | 0,3716 | | 0,53 | 0,31 |
| 130 | | | 0,5151 | | 0,51 | 0,64 |
| 138 | | | 0,6878 | | 0,81 | 1,66 |
| 146 | | | 0,4198 | | 0,49 | 0,94 |
| 3 | | | 0,6688 | | 1,19 | 1,92 |
| 21 | | | 0,7674 | | 0,15 | 0,27 |
| 117 | Female | Early vit. WT | 0,4600 | | 0,24 | 0,69 |
| 120 | | | 0,4694 | | 0,17 | 0,37 |
| 121 | | | 0,1103 | | 0,2 | 0,52 |
| 127 | | | 0,3499 | | 0,13 | 0,54 |
| 185 | | | 0,7833 | | 1,85 | 1,99 |
| 59 | | | 0,4818 | | 0,21 | 0,63 |
| 61 | | | 0,6475 | | 0,53 | 1 |
| 78 | | | 0,3578 | | 0,33 | 0,72 |
| 4 | | | 0,0277 | 0,1 | | |
| 6 | | | 0,0267 | 0,77 | | |
| 14 | | | 0,0288 | 0,14 | | |
| 15 | | | 0,0184 | 0,23 | | |
| 18 | | | 0,0339 | 0,41 | | |
| 19 | | | 0,0293 | 0,75 | | |
| 54 | | | 0,0283 | 1,23 | | |
| 67 | | | 0,0265 | 0,58 | | |
| 89 | | | 0,0168 | 0,47 | | |
| 96 | | | 0,0414 | 1,03 | | |
| 97 | Male | GCF | 0,0086 | 0,82 | | |
| 98 | | | 0,0293 | 0,26 | | |
| 104 | | | 0,0292 | 0,02 | | |
| 105 | | | 0,0339 | 0,37 | | |
| 106 | | | 0,0218 | 0,71 | | |
| 107 | | | 0,0231 | n.d. | | |
| 110 | | | 0,0449 | 0,02 | | |
| 111 | | | 0,0702 | 0,43 | | |
| 114 | | | 0,0174 | n.d. | | |
| 116 | | | 0,0379 | 0,3 | | |
| 122 | | | 0,0193 | 0,36 | | |

| | | | | | |
|---|---|---|---|---|---|
| 125 | | | 0,0391 | 0,02 | |
| 139 | | | 0,0464 | 0,69 | |
| 148 | | | 0,0209 | n.d. | |
| 152 | | | 0,0294 | n.d. | |
| 153 | | | 0,0232 | 0,14 | |
| 157 | | | 0,0240 | n.d. | |
| 181 | | | 0,0157 | 0,42 | |
| 1 | | | 0,0735 | 0,28 | |
| 7 | | | 0,1894 | 0,53 | |
| 9 | | | 0,0536 | 0,26 | |
| 22 | | | 0,2084 | 0,64 | |
| 25 | | | 0,0833 | 0,64 | |
| 33 | | | 0,0931 | 0,42 | |
| 55 | | | 0,2396 | 3,68 | |
| 57 | Male | Immature WT | 0,0898 | 1,57 | |
| 74 | | | 0,0373 | 0,56 | |
| 91 | | | 0,1433 | 2,65 | |
| 92 | | | 0,0530 | 1,06 | |
| 131 | | | 0,0485 | 0,67 | |
| 158 | | | 0,0661 | 0,42 | |
| 170 | | | 0,2490 | 1,86 | |
| 178 | | | 0,1374 | n.d. | |
| 11 | | | 1,3456 | 44,75 | |
| 20 | | | 1,2944 | 71,68 | |
| 23 | | | 2,2563 | 21,97 | |
| 29 | | | 3,9699 | 44,96 | |
| 38 | | | 0,8102 | 25,87 | |
| 49 | | | 3,4783 | | |
| 50 | | | 4,1014 | 28,92 | |
| 60 | | | 3,3366 | 58,91 | |
| 65 | | | 1,8302 | 48,04 | |
| 68 | | | 4,6854 | 75,69 | |
| 69 | | | 1,2710 | 54,08 | |
| 70 | | | 6,5228 | 22,8 | |
| 82 | | | 5,4600 | 44,47 | |
| 85 | | | 1,0036 | 44,46 | |
| 86 | | | 1,2446 | 51,77 | |
| 99 | Male | Mature WT | 2,5675 | 49,54 | |
| 100 | | | 3,1616 | 101,77 | |
| 101 | | | 4,5932 | 1,46 | |
| 102 | | | 3,3488 | n.d. | |
| 103 | | | 1,1628 | n.d. | |
| 109 | | | 5,4677 | 73,83 | |
| 124 | | | 2,0877 | 50,41 | |
| 126 | | | 2,9150 | | |
| 128 | | | 0,8043 | 21,29 | |
| 140 | | | 3,5257 | 47,67 | |
| 144 | | | 3,6192 | 52,15 | |
| 149 | | | 4,2403 | 53,15 | |
| 155 | | | 4,2870 | 85,53 | |
| 172 | | | 0,9564 | 45,21 | |
| 159 | | | 4,1004 | 71,45 | |
